# Prevalence and associated factors of cancer pain among adult cancer patients evaluated at an oncology unit in the University of Gondar Comprehensive Specialized Hospital, northwest Ethiopia

**DOI:** 10.3389/fpain.2022.1061239

**Published:** 2023-02-17

**Authors:** Anteneh Ayelign Kibret, Haileab Fekadu Wolde, Abebe Muche Moges, Hailu Aragie, Ephrem Tafesse Teferi, Yohannes Awoke Assefa, Endalkachew Belayneh Melese, Mequanint Melesse, Yilkal Belete Worku, Daniel Gashaneh Belay, Meseret Derbew Molla, Dagnew Getnet Adugna

**Affiliations:** ^1^ Department of Human Anatomy, School of Medicine, College of Medicine and Health Sciences, University of Gondar, Gondar, Ethiopia; ^2^ Department of Epidemiology and Biostatistics, Institute of Public Health, College of Medicine and Health Sciences, University of Gondar, Gondar, Ethiopia; ^3^ Department of Internal Medicine School of Medicine, College of Medicine and Health Sciences, University of Gondar, Gondar, Ethiopia; ^4^ Department of Occupational Therapy School of Medicine, College of Medicine and Health Sciences, University of Gondar, Gondar, Ethiopia; ^5^ Department of Obstetrics and Gynecology School of Medicine, College of Medicine and Health Sciences, University of Gondar, Gondar, Ethiopia; ^6^ Department of Biochemistry, School of Medicine, College of Medicine and Health Sciences, University of Gondar, Gondar, Ethiopia

**Keywords:** associated factors, University of Gondar, cancer pain, prevalence, Ethiopia

## Abstract

**Introduction:**

Globally, cancer is the second leading cause of death and was responsible for 9.6 million deaths in 2018. Worldwide, 2 million people experience pain every day, and cancer pain is one of the major neglected public health problems, especially in Ethiopia. Despite reporting the burden and risk factors of cancer pain as a principal importance, there are limited studies. Therefore, this study aimed to assess the prevalence of cancer pain and its associated factors among adult patients evaluated at the oncology ward in the University of Gondar Comprehensive Specialized Hospital, northwest Ethiopia.

**Methods:**

An institution-based cross-sectional study was conducted from 1 January to 31 March 2021. A systematic random sampling technique was used to select the total sample size of 384 patients. Data were collected using pretested and structured interviewer-administered questionnaire. Bivariate and multivariate logistic regression models were fitted to identify the factors associated with cancer pain among patients with cancer. An adjusted odds ratio (AOR) with a 95% CI was computed to determine the level of significance.

**Results:**

A total of 384 study participants were involved, with a response rate of 97.5%. The proportion of cancer pain was found to be 59.9% (95% CI 54.8–64.8). The odds of cancer pain were escalated by anxiety (AOR = 2.52, 95% CI 1.02–6.19), patients with hematological cancer (AOR = 4.68, 95% CI 1.30–16.74), gastrointestinal cancer (AOR = 5.15, 95% CI 1.45–18.2), and stages III and IV (AOR = 14.3, 95% CI 3.20–63.7).

**Conclusion:**

The prevalence of cancer pain among adult patients with cancer in northwest Ethiopia is relatively high. Variables such as anxiety, types of cancer, and stage of cancer had a statistically significant association with cancer pain. Hence, to advance the management of pain, it is better to create more awareness regarding cancer-related pain and provide palliative care early on in the diagnosis of the disease.

## Introduction

Cancer is the second leading cause of death globally and is responsible for 9.6 million deaths in 2018; approximately 70% of deaths occurred in low-income countries (1). Cancer is becoming an increasing public health burden in Ethiopia and sub-Saharan Africa at large. The Federal Ministry of Health (FMOH) estimated that more than 150,000 cases of cancer were diagnosed each year and account for 4% of all deaths (2). Throughout their clinical course, patients with cancer experience various symptoms. One of the most dreaded symptoms is pain; this could be due to either the cancer itself and/or the cancer treatment (3, 4). Patients with advanced or metastatic cancer more frequently experience cancer-related pain (5). It is a major source of suffering that requires special treatment and care. Therefore, pain management is found to be an essential part of oncology care. The WHO estimates that 80% of the world population has insufficient access to appropriate opioid analgesics, such as morphine (6, 7). Despite the presence of guidelines for the management of cancer pain, many patients with cancer are still under treatment. There are also various forms of barriers towards the inadequate management of cancer-related pain, such as the inappropriate use and fear of opioids due to patients' cultural attitudes regarding pain and the use of opioid medications (8–10). A recently conducted study revealed that 2 million people in the world experience cancer pain every day; however, it is still one of the major neglected public health problems in low-income countries, especially in Ethiopia. In oncology patients, the prevalence of cancer-related pain is estimated to be up to 25%, 33%, and more than 75% in newly diagnosed patients, those who are under active treatment, and in patients with advanced stages of cancer, respectively (11). According to studies conducted in some African countries, the prevalence of cancer pain was in the range of 35.7%–91.6% (11–13). In addition to physical pain, patients with cancer are prone to psychological problems, such as anxiety and depression. These problems are usually related to the reactions of a cancer diagnosis, cancer type, treatment effects, recurrence, fear of end-of-life, survivorship, and financial burden (14, 15). Furthermore, factors such as age, sex, marital status, educational status, occupation, income, type of cancer, stage of the diseases, length of stay before the treatment, and duration of the treatment presumably aggravate cancer-related pain (10, 16). Studies on the assessment of the prevalence of cancer pain and its associated factors in Ethiopia are very limited. Therefore, the aim of the present study was to assess the prevalence of cancer pain and its associated factors among adult patients evaluated at the oncology ward, University of Gondar Comprehensive Specialized Hospital (UoGCSH). The results of this study will be helpful in raising the awareness of policy makers, healthcare mangers, and healthcare professionals about cancer-related pain. This will subsequently pave the way to provide comfort to patients with cancer and improve and strengthen preventive strategies for the management of cancer pain. Moreover, the results of the study can be used as baseline data for further related studies.

## Materials and methods

### Study design and period

An institution-based cross-sectional study was conducted at UoGCSH from 1 January to 31 March 2021 among adult patients with cancer evaluated in the oncology ward. UoGCSH is located in northwest Ethiopia, 750 km from the capital. It is a teaching hospital that serves a total population of around 5 million. It provides clinical services in different departments, including internal medicine, surgery, gynecology and obstetrics, and pediatrics. In addition, the hospital has both pediatric and adult oncology wards. The ward serves more than 3,000 patients with cancer every year. The oncology unit currently has 32 beds for the treatment of patients with cancer.

### Study population and sample size determination

The source population of the present study was all adult patients with cancer who visited the outpatient and inpatient oncology departments at the UoGCSH. All adult patients with cancer who visited the oncology center between 1 January and 31 March 2021, and who were available during the time of data collection, made up the study population. However, adult cancer patients who were unable to communicate and/or who had severe psychiatric problems were excluded from the study. The sample size for the prevalence and for the associated factors was determined using a single population proportion formula and the power approach, respectively, and we determined a sample size of 342. Finally, after adding 15% as a non-response rate, the sample size was found to be 394 and participants were selected using a systematic random sampling technique with a skip interval of two.

### Variables and data collection procedure

The outcome variable of this study was cancer pain, and it was assessed using items 2–5 of the Brief Pain Inventory-Short Form (BPI-sf) (17). Patients were asked to grade their worst, least, and average pain in the last week and the pain they felt currently. The scoring for each item was in the range of 0–10: 0 indicates no pain and 10 indicates the most severe form of the pain, which is explained as “Pain as bad as you can imagine.” The pain severity score was then calculated by adding the scores from the four items and dividing it by four (18); the final was classified as 0 (no pain), 1–3 (mild pain), 4–7 (moderate pain), and 8–10 (severe pain). The first group of factors assessed were sociodemographic characteristics, including age, sex, residence, occupation, marital status, and average monthly income. The second group was clinical factors that included type of cancer, cancer stage, site of the cancer, time since diagnosis, presence of co-morbidities (HIV, diabetes mellitus, hypertension), type of cancer treatment, presence of metastasis, and length of stay before starting treatment. The third group of characteristics assessed were behavioral and psychosocial factors and mainly focused on smoking, alcohol intake, and anxiety.

Data were collected using a pretested and structured interviewer-administered questionnaire from the chart reviews of cancer patients. Information related to cancer pain and adequacy of treatment was collected using the BPI-sf (17), which consists of eight items. The first item is to identify where they felt pain and items 2–4 are to assess the severity of the pain. Items 6 and 7 are to assess the type of analgesics used and the adequacy of pain management, and item 8 is to measure the effect of the pain on daily activities. Physical activity was assessed according to WHO steps, by which any movement of the body produced by skeletal muscle, which requires energy expenditure, was taken as physical activity. Thus, physical activity was categorized into three levels: vigorous; moderate; and inadequate or poor. A vigorous-intensity activity was defined as any activity that causes a large increase in breathing or heart rate (e.g., running, carrying, or lifting heavy loads, digging or construction work) that continues for at least 30 min for a minimum of 3 days per week. Moderate-intensity activity was defined as any activity that causes a small increase in breathing or heart rate (brisk walking or carrying light loads) that continues for at least 30 min for at least 3 days per week, or 5 or more days of these activities for at least 20 min per day, or ≥3 days of vigorous-intensity activity per week for at least 20 min per day. Low-level (sedentary) physical activity was defined as an individual having physical activity that does not meet any of these criteria (19). Anxiety was measured using the Generalized Anxiety Disorder 7-item (GAD-7) scale. The GAD-7 questionnaire is a brief measure of generalized anxiety disorder that assesses problems that bothered the respondent in the past 2 weeks. The items measure the frequency of symptoms on a scale from 0 (not at all) to 3 (nearly every day) (20). Information on the variables, such as type and stage of cancer, type of treatment, and type of analgesics, was collected from patient charts.

The data were collected by three physicians working in the oncology ward; data collection was supervised by the principal investigator. The data were checked for any inconsistencies, coding errors, values out of range, completeness, accuracy, clarity, and missing values; appropriate corrections were made consistently by the principal investigator on a daily basis.

### Data processing and analysis

The data processing and analysis section has been described in a previously published article (21).

## Results

### Sociodemographic characteristics

In the present study, a total of 384 patients with cancer were involved, with a response rate of 97.5%. The mean age of the participants was 49 ± 13 years. Of the study participants, 197 (51.3%) were women and more than half of the study participants (*n*=246, 64.4%) were from rural areas. Of the participants, 144 (37.5%) were farmers and 191 (40.7%) were married. The majority of the study participants (*n*=249, 64.8%) had an average monthly income in the range of 1,211–8,970 ETB (Table 1).

**Table 1:** Socio-demographic characteristics of adult cancer patients evaluated at oncology unit in UoGCSH, Northwest Ethiopia, 2,021 (*n* = 384).

Variables	Frequency	Percentage
Sex		
Male	187	48.7
Female	197	51.3
Age		
18–38	73	19.01
39–59	219	57.03
60–82	92	23.96
Residence		
Urban	136	35.6
Rural	246	64.4
Occupation		
Farmer	144	37.5
Merchant	82	21.35
Government employee	119	30.95
Student	13	3.39
Others^a^	26	6.77
Religion		
Orthodox	243	63.28
Muslim	91	23.70
Protestant	45	11.72
Catholic	5	1.30
Marital status		
Married	191	49.74
Divorced	73	19.01
Widowed	76	19.79
Single	44	11.46
Average monthly income in ETB		
<1,210	112	29.17
1,211–8,970	249	64.84
>8,971	23	5.99

^a^ Unemployed, solider, drivers, retire, daily laborers, and artist.

### Clinical and behavioral characteristics

Of the patients with cancer, most of them (87.2%) developed anxiety and more than one-third (36.2%) had poor social support. More than half of the patients (52.6%) did poor physical exercise and most of the patients (58.07%) were found in the first stage of the disease. Gynecological-related cancer (17.97%) and hematological cancer (17.71%) had an almost equal proportion and were the most common types of cancer. Regarding treatment modalities, of the participants who took treatment, 124 (32.2%) and 111 (28.9%) underwent surgery and a combination therapy (surgery and chemotherapy), respectively (Table 2).

**Table 2:** Clinical and behavioral characteristics of adult cancer patients evaluated at oncology unit in UoGCSH, Northwest Ethiopia, 2021 (*n* = 384).

**Variable**	**Frequency**	**Percentage**
**Psychosocial and behavioral factors**		
** Alcohol drink**		
** Non-alcoholic**	183	47.66
** Alcoholic**	201	52.34
** Cigarette smoking**		
** Non-smoker**	322	83.85
** Smoker**	62	16.15
** Chat chewing**		
** Not chat used**	281	73.18
** Chat used**	103	26.82
**Physical exercise**		
** Vigorous**	50	13.02
** Moderate**	132	34.38
** Poor**	202	52.6
**Anxiety**		
** No**	49	12.76
** Yes**	335	87.24
**Social support**		
** Poor**	139	36.2
** Moderate**	188	48.96
** Strong**	57	14.84
**Medical-related conditions**		
** Types of CA**		
** Lung**	16	4.17
** Breast**	55	14.32
** Gynecological**	69	17.97
** Hematologic**	64	16.67
** GI**	68	17.71
** Skin**	15	3.91
** GU**	25	6.51
** Endocrine**	36	9.38
** Other^a^**	36	9.38
** Stages**		
** Stage I**	223	58.07
** Stage II**	70	18.23
** Stage III**	52	13.54
** Stage IV**	39	10.6
** Metastasis**		
** No**	265	69.01
** Yes**	119	30.99
** Length of stay before treatment**		
** <4 months**	289	90.88
** ≥4 months**	29	9.12
** Treatment modality**		
** No treatment**	61	15.89
** Chemotherapy**	88	22.92
** Surgery**	124	32.29
** Combination**	111	28.91
** Chronic diseases**		
** Absent**	245	63.80
** Present**	139	36.20
** Anti-pain**		
** Yes**	226	58.9
** No**	158	41.1

^a^ Cancer types such as osteogenic, eye, soft tissue sarcoma, and neck carcinoma.

### Prevalence of cancer pain

The prevalence of cancer pain among adult patients with cancer at the UoGCSH, northwest Ethiopia, was 59.9% (95% CI 54.8–64.8). It was more prevalent (86.1%) among patients with stage III and IV cancer, who had anxiety (63%) and metastasis (76.4%). Of the patients with cancer pain, 157 (68.2%), 29 (12.6%), and 44 (19.1%) experienced mild, moderate, and severe pain, respectively. The highest proportion of cancer pain was seen in patients with gastrointestinal (GI) cancer (*n*=55, 30%) followed by those with hematologic cancer (*n*=48, 20.8%) (Table 3).

**Table 3:** The proportion of cancer pain in each cancer type among patients attending the oncology unit of UoGCSH, Northwest Ethiopia 2021 (*n* = 230).

Cancer types	Cancer pain in frequency	Percentage
Lung	9	2.72
Breast	42	18.2
Gynecological	24	10.4
Hematologic	48	20.8
GI	55	30
Skin	8	3.47
GU	12	5.2
Endocrine	18	7.8
Other^a^	14	6.08
Total	230	100.0

^a^ Cancer types such as osteogenic, eye, soft tissue sarcoma and neck carcinoma.

### Factors associated with cancer pain

From the variables treated using a multivariate analysis, having anxiety, types of cancer, and stage of cancer had a significant association with the occurrence of cancer pain.

In the present study, patients who had anxiety had a 2.5 times higher chance of having cancer pain than those who did not have anxiety (AOR = 2.52, 95% CI 1.02–6.19). The odds of having cancer pain were 4.6 times higher among patients with hematological cancer compared to other types of cancer (AOR = 4.68, 95% CI 1.30–16.74). The odds of having cancer pain were 5.1 times higher among patients with GI cancer compared to other types of cancer (AOR = 5.15, 95% CI 1.45–18.2). Patients with stage III and IV of cancer were 14.3 times more likely to have cancer pain compared to those patients with stage I cancer (AOR = 14.3, 95% CI 3.20–63.7) (Table 4).

**Table 4:** Bivariable and multivariable analyses of factors associated with cancer pain among patients in the UoGCSH, Northwest Ethiopia 2021.

**Variables**	**Categories**	**Cancer pain**		**COR [95% CI]**	**AOR [95% CI]**
		**Yes (%) *n* = 230 (59.64)**	**No (%) *n* = 155 (40.36)**		
**Sex**	Male	118	69	1.00	1.00
	Female	112	85	0.77[0.51, 1.16]	1.18 [0.55, 2.65]
**Anxiety**	No	19	30	1.00	1.00
	Yes	211	124	2.68 [1.45, 4.97]	2.52 [1.02, 6.19]*
**Length of stay before treatment**	<4 months	182	107	1.00	1.00
	≥4 months	16	13	0.72 [0.33, 1.56]	1.37 [0.54, 3.43]
**Occupation**	Farmer	92	52	0.75 [0.31, 1.51]	0.48 [0.15, 1.48]
	Merchant	46	36	0.50 [0.22, 1.14]	0.44 [0.13, 1.50]
	Government employees	64	55	0.45 [0.20, 1.00]	0.45 [0.14,1.38]
	Other	28	11	1.00	1.00
**Types of cancer**	Lung	9	7	2.02 [0.61, 6.67]	5.35 [0.70, 40.77]
	Breast	42	13	5.07 [2.03, 12.7]	4.01[0.98., 16.4]
	Gynecological	24	45	0.83 [0.36, 1.92]	0.43 [0.11, 1.70]
	Hematologic	48	16	4.71 [1.96, 11.3]	4.68 [1.30, 16.74]*
	GI	55	13	6.64 [2.69, 16.3]	**5.15 [1.45, 18.2]** *
	Skin	8	7	1.79 [0.53, 6.05]	3.78 [0.56, 24.02]
	GU	12	13	1.45 [0.51, 4.07]	1.10 [0.25, 4.79]
	Endocrine	18	18	1.45 [0.61, 4.00]	1.20 [0.30, 4.73]
	Other*	14	22	1.00	1.00
**Stage of cancer**	Stage I	110	110	1.00	1.00
	Stage II	39	31	1.25 [0.73, 2.16]	2.18 [0.93, 5.10]
	Stage III and IV	81	13	6.23[3.27, 11.8]	14.3 [3.20, 63.71]**
**Metastasis**	Yes	91	28	2.94 [1.80, 4.79]	0.44[0.12, 1.53]
	No	139	126	1.00	1.00
**Chronic diseases**	Absent	144	101	1.00	1.00
	Present	86	53	1.13 [0.74, 1.74]	1.54 [0.84, 2.82]

AOR, adjusted odds ratio; COR, Crude odds ratio; CI, confidence interval.

* *P*-value <0.05, **P-value <0.01, ***P-value <0.001.

## Discussion

Pain is one of the most frequent and distressing symptoms experienced by patients with cancer, affecting their quality of life and causing serious health problems throughout the world. The aim of this study was to assess the prevalence and associated factors of cancer pain among adult patients with cancer at the UoGCSH, northwest Ethiopia. Our findings revealed that the prevalence of cancer-related pain was 59.9% (95% CI 54.8–64.8). This result is consistent with a study conducted in the Netherlands (55%) (16), but was higher than in studies conducted in South Africa (35.7%) and the United States (29.8%) (12, 22). However, it is lower than in studies conducted in the same setting in 2017 and in Addis Ababa, Ethiopia (10, 23). This discrepancy may be due to an inconsistent level of attention for cancer pain across the world. In low-income countries such as Ethiopia, physicians have given less attention to the management of pain among patients with cancer, or they mainly focused on the treatment of the patients. The major causes of cancer-related pain are lack of physicians’ and patients’ knowledge, lack of adequate supply of pain relievers, poverty, and illiteracy (5, 24). Thus, the finding of cancer pain in this study is much higher than in similar studies conducted in high-income countries, such as South Africa and the United States. This might be due to the physicians in high-income countries focusing equally on both the disease and pain management. On the contrary, the prevalence rate of cancer pain in the current study is lower than in the studies conducted in Gondar and Addis Ababa. This might be due to the recently increased number of oncologists in our country who may pay more attention to the management of cancer pain.

In the present study, patients who had anxiety had a higher chance of experiencing cancer pain than their counterparts. This is supported by a study conducted in Addis Ababa, Ethiopia (25). Anxiety can create its own kind of pain by tensing up the muscles, which can lead to pain and stiffness in almost any area of the body. Constant stress and worry can also suppress the normal activity of the immune system, leading to decreased resistance to infection and disease. Infection increases inflammation in the body, which can cause a range of symptoms, including joint pain. Anxiety might also increase the occurrence of pain through the release of catecholamines, which are peripherally sensitizing or stimulating nociceptors (25).

In our study, the odds of having cancer pain were higher among patients with hematological and GI cancer compared to patients with other types of cancer. This finding is supported by a study conducted in the Netherlands (16). In patients with hematological cancer, the disease often involves the bone marrow and forms a skeletal lesion. This lesion then generates localized and/or irradiated nociceptive continuous pain at rest, sometimes complicated by neuropathic symptoms (mixed pain) and pain from movement-related incidents. In addition, onco-hematological patients are highly predisposed to painful infections, such as pneumonia, cellulitis, urinary tract infections, wound infections, oral and GI mucositis, esophagitis by *Candida*, oral and genital herpes, herpes zoster, and postherpetic neuralgia (26, 27). In addition, GI cancers, such as colorectal, stomach, and pancreatic cancer, are usually associated with pain due to the spread of the tumor towards the lower spine, which further leads to spinal cord compression. Further, it is characterized by intense tumor pressure on the spinal cord, which in turn causes severe lower back pain (28). The association of pain and GI cancers may also be explained due to the frequent requirement of surgery for the management of the cancer. Most of the GI cancers may spread into the intestines, which leads to excruciating bowel obstructions that require surgery. If postoperative care is not accurately performed, surgery-associated cancer pain can considerably occur with GI cancers compared to other cancer types that do not require surgery (29, 30).

In the present study, patients with stage III and IV cancer had more cancer pain compared to patients with stage I cancer. This is supported by a study in Addis Ababa, Ethiopia and the Netherlands. Studies have shown that patients with advanced cancer are more likely to have complications, such as pain (10, 16, 22). The more advanced the stage of cancer, the more the patient is likely to experience cancer pain. A possible reason is as the cancer becomes more advanced, it starts to involve many structures, organs, or systems through its secondary spread. In addition, cancer progression may result in tissue damage and/or nerve injury through various mechanisms, such as infiltration, obstruction, compression, and fracture, leading to the exacerbation of cancer-related pain (31). Therefore, physicians should focus their attention on the management of cancer pain, particularly in those patients who are in the advanced stages of cancer, those experiencing anxiety, and those with hematologic and GI cancers.

The present study has several strengths, including the adequate sample size that empowered the study; data were collected by physicians who were working at the oncology unit. Moreover, the study comprises all adult patents with all types of cancer and stages. Since our study used a cross-sectional study design, it could not establish a cause–effect relationship and there could be a possibility of recall bias. In addition, the study did not specify the site of metastasis; rather, it indicated the presence or absence of metastasis. In our study, we considered all the classifications of hematological malignancy (acute lymphocytic, chronic lymphocytic, acute myeloid, chronic myeloid, myeloma, and lymphoma [Hodgkin's and non-Hodgkin's]), which means that patients with any of these classifications were considered to have hematologic malignancy in general.

## Conclusion

The prevalence of cancer pain among adult patients with cancer in northwest Ethiopia is relatively high. Variables, such as anxiety, types of cancer, and stage of cancer, had a statistically significant association with cancer pain. More awareness about cancer-related pain is needed to improve pain management and encourage the referral to palliative care early in the diagnosis of the disease. After the diagnosis of cancer, the patients should be approached and evaluated by psychiatrists to reduce the occurrence of anxiety. A further prospective follow-up study should be conducted.

## Data Availability

The raw data supporting the conclusions of this article will be made available by the authors, without undue reservation.
